# Nucleic Acid Vaccines against SARS-CoV-2

**DOI:** 10.3390/vaccines10111849

**Published:** 2022-10-31

**Authors:** Ying Liu, Qing Ye

**Affiliations:** Department of Clinical Laboratory, The Children’s Hospital, Zhejiang University School of Medicine, National Clinical Research Center for Child Health, National Children’s Regional Medical Center, Hangzhou 310052, China

**Keywords:** nucleic acid vaccines, COVID-19, development process, advantages and shortcomings, optimization

## Abstract

The coronavirus disease 2019 (COVID-19) has spread worldwide and imposed a substantial burden on human health, the environment, and socioeconomic development, which has also accelerated the process of nucleic acid vaccine development and licensure. Nucleic acid vaccines are viral genetic sequence-based vaccines and third-generation vaccines after whole virus vaccines and recombinant subunit vaccines, including DNA vaccines and RNA vaccines. They have many unique advantages, but there are many aspects that require optimization. Therefore, the purpose of this review is to discuss the research and development processes of nucleic acid vaccines, summarize the advantages and shortcomings, and propose further optimization strategies by taking COVID-19 vaccines as an example. Hopefully, this work can make a modest contribution in promoting the construction of emergency nucleic acid vaccine platforms and in avoiding the reemergence of similar public health emergencies.

## 1. Introduction

Coronaviruses (CoVs) are widespread in nature and can cause multisystem disorders in humans [1,2,3,4,5,6], including the respiratory and alimentary tract, nervous system, etc., and lead to immense financial loss at the same time [7]. To date, CoV infections, including severe acute respiratory syndrome coronavirus 2 (SARS-CoV-2), have resulted in three global pandemics [8,9,10,11]. Due to its greater ability to recognize receptors [12], SARS-CoV-2 spreads more efficiently from person to person [13,14]. Currently, drug development is side-by-side with vaccine upgrading. Vaccination is the most effective strategy for preventing and controlling infectious diseases [15,16,17,18]. Multiple types of SARS-CoV-2 vaccines have been developed at the same time [19], and some of them have successively been granted emergency use authorization by the World Health Organization (WHO), mainly including inactivated vaccines [20,21], adenovirus-vectored vaccines [22,23], and nucleic acid vaccines [24,25]. Due to well-established technology, vaccination with inactivated vaccines is the primary method used during our epidemic prevention and control mechanisms. However, vaccination with inactivated vaccines, in most cases, result in the generation of humoral, but not cell-mediated, immune responses. With the deepening research of virology and the gradual maturation of vaccine technology, substantial progress has been made in the development and application of nucleic acid vaccines, which, as an emerging platform, have become a hotspot in the vaccine research and development field.

Nucleic acid vaccines, as an emerging concept, were established in the early 1990s [26] and include DNA vaccines and RNA vaccines, which were also third-generation vaccines after whole virus vaccines and recombinant subunit vaccines. After introducing foreign target genes, they use the protein synthesis systems of host cells to express target proteins and then induce immune responses. Before the outbreak of coronavirus disease 2019 (COVID-19), nucleic acid vaccines were not yet available for human use on the market. The unprecedented pandemic scenario has accelerated the vaccine development and licensure process. The purpose of this review is to discuss the research and development processes of nucleic acid vaccines, summarize the advantages and shortcomings, and propose further optimization strategies by taking COVID-19 vaccines as an example. Hopefully, this work can make a modest contribution in promoting the construction of emergency nucleic acid vaccine platforms and in avoiding the reemergence of similar public health emergencies.

## 2. The Research and Development Process of Nucleic Acid Vaccines

**Figure 1:**
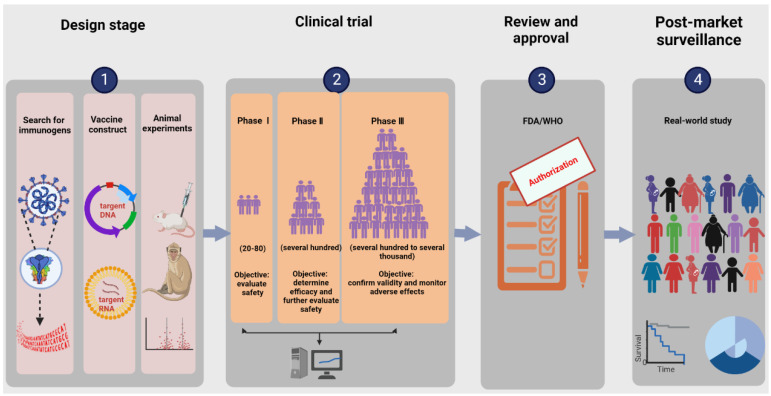
Illustration of the research and development process for nucleic acid vaccines. The procedure involves an early design stage, clinical trial stage, review and approval stage, and post-market surveillance. The early design stage generally consists of searching for immunogens, designing vaccine structures, and determining toxicological effects and immune effects in animal models. The clinical trials mainly include phases Ⅰ–Ⅲ, targeting primarily practical applications to provide definitive evidence for the safety and efficacy of vaccines.

The research and development procedure of nucleic acid vaccines involves two main phases: early design stage and clinical experiment stage. More details are shown in Figure 1. The early design stage generally consists of searching for immunogens, designing vaccine structures, and determining toxicological effects and immune effects in animal models. The clinical experiments targeting primarily practical application mainly aim to provide definitive evidence for the safety and efficacy of vaccines. According to regulations of special approval processes, on the premise of guaranteeing the security and stability of COVID-19 vaccines, it is admissible to reduce certain approval processes accordingly [27]. Additionally, the vaccine life cycle includes production, supply, available on the market, and post-marketing research in the real world. Currently, the ZyCoV-D vaccine developed by Cadila in Ahmedabad, Gujarat, India is the first DNA vaccine for people to be approved anywhere in the world [28]. INO-4800, developed by Inovio (the leading global development corporation of DNA vaccines), is the first DNA vaccine to advance to clinical trials and is currently undergoing phase three clinical trials, having the prospect of being commercially available within one year. BioNTech/Pfizer and Moderna are two leading research teams for COVID-19 RNA vaccines. BNT162b2 from BioNTech/Pfizer and mRNA 1273 from Moderna were granted emergency use authorization by the WHO on 14 January 2021 and on 3 February 2021, respectively. An illustration of the current COVID-19 nucleic acid vaccines is presented in Table 1.

### 2.1. Search for Immunogens

Searching for appropriate immunogens marks the first step toward the development of nucleic acid vaccines. Similar to other CoVs, SARS-CoV-2 has a typical structure comprising a nucleocapsid and an envelope. The envelopes are studded with the spike protein (S), envelope protein (E), and membrane protein (M), which wrap the nucleocapsid encapsulating single, positive-stranded RNA [29,30]. The E and M proteins are mainly involved in the processing of viral assembly, while the S protein mediates membrane fusion and viral entry. When viruses fuse with host cell membranes, S proteins undergo dramatic structural rearrangement. The S protein is composed of S1 and S2 subunits. S1, the N-terminal furin cleavage fragment, contains the receptor-binding domain (RBD) and is used to recognize and bind to cells expressing angiotensin-converting enzyme 2 (ACE2) receptors in humans [31], while S2, the C-terminal furin cleavage fragment, contains the fusion machinery [32]. The overexpression of human ACE-2 receptors enhanced disease severity and lung injury in a transgenic mouse model [33]. Together, the S protein is a major antigenic determinant and an important target for vaccine development, playing a critical role in mediating viral entry.

### 2.2. Designing Vaccine Constructs

**Figure 2:**
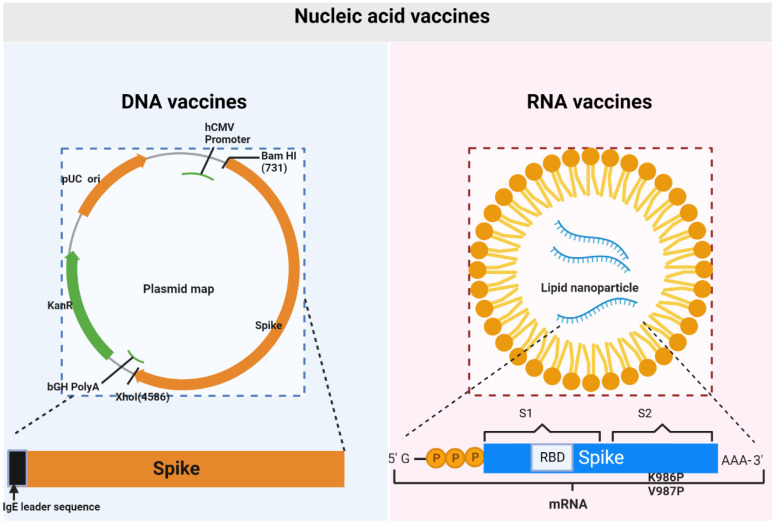
Schematic diagram of COVID-19 synthetic DNA and RNA vaccine constructs. Schematic diagram of COVID-19 synthetic DNA vaccine constructs (**left**), where plasmids containing the IgE leader sequence and SARS-CoV-2 spike protein insert are shown. Schematic diagram of COVID-19 synthetic RNA vaccine constructs (**right**), showing the S1 (N-terminal furin cleavage fragment), S2 (C-terminal furin cleavage fragment), and RBD (receptor-binding domain). The positions of the P2 mutation (K986P and V987P) are indicated.

Vaccine constructs are central in determining the success or failure of a new vaccine. Nucleic acid vaccines are viral genetic sequence-based vaccines (COVID-19 vaccines are S protein genetic sequence-based). The Chinese Center for Disease Control and Prevention released the SARS-CoV-2 genetic sequence and disseminated it globally by the GISAID (Global Initiative on Sharing All Influenza Data) initiative on 11 January 2020 [13]. Thereafter, the development of nucleic acid vaccines was initiated. A DNA vaccine is a vaccine that directly transfects recombinant plasmids (the circular strands of DNA) containing target DNA sequences into the host cell nucleus to overexpress and then induce antigen-specific immune responses. An RNA vaccine is a novel type of vaccine and is composed of mRNA (synthesized efficiently from DNA templates by in vitro transcription) packaged within a vector [34,35], such as lipid nanoparticles, polymeric carriers, protamine, and dendritic cells, which can advance protein translation and posttranslational modification in the cytoplasm directly. A schematic diagram of COVID-19 synthetic DNA and RNA vaccine constructs is shown in Figure 2. In addition to nucleic acid components, complete nucleic acid vaccines also include vectors and adjuvants, all of which affect the safety and efficacy of vaccination to varying degrees.

For ZyCoV-D, synthesis of the S protein gene containing the IgE signal peptide gene region and further cloning into the pVAX-1^®^ vector (Thermo Fisher Scientific, Shanghai, China) resulted in the generation of a vaccine construct. Restriction digestion with BamHI—resulting in linearized DNA fragments of ~6.78 kb—and restriction digestion analysis with NheI and ApaI—resulting in the generation of fragments of ~2.89 kb of vector and ~3.89 kb of S gene—have been used to confirm the insertion of S into the vector [36]. For INO-4800, the specific name of the plasmid is pGX9501, subsequently termed INO-4800, with the highly optimized DNA sequence encoding SARS-CoV-2 IgE-S (add the N-terminal IgE leader sequence). The optimized DNA sequence was created using Inovio’s proprietary in silico Gene Optimization Algorithm to enhance expression and immunogenicity. The optimized DNA sequence was synthesized, digested with BamHI and XhoI, and cloned into the expression vector pGX0001 under the control of the human cytomegalovirus immediate-early promoter and a bovine growth hormone polyadenylation signal [37].

For RNA vaccines of BNT162b2 and mRNA-1273, the mRNA is purified by oligo-dT affinity purification and encapsulated in a lipid nanoparticle through a modified ethanol-drop nanoprecipitation process [38]. The encapsulation of lipids allows the mRNA to transfect into host cells efficiently after intramuscular injection. To model the complete antigen structure, they all encode membrane-anchored prefusion protein S and stabilize the prefusion state by mutating the S residues 986 and 987 to prolines [39,40,41]. The excipients of BNT162b2 also include ALC-0315, ALC-0159 (polyethylene glycol), cholesterol, potassium chloride, potassium dihydrogen phosphate, sodium chloride, disodium hydrogen phosphate dihydrate, sucrose, and water for injection [25]. The mRNA-1273 vaccine also contains the following ingredients: lipids (SM-102, 1,2-dimyristoyl-rac-glycero-3-methoxypolyethylene glycol-2000 (PEG2000-DMG), cholesterol, and 1,2-distearoyl-sn-glycero-3-phosphocholine (DSPC)), tromethamine, tromethamine hydrochloride, acetic acid, sodium acetate, and sucrose [24].

Prior to vaccination in animal models, the expression of target antigens should be confirmed in cells in vitro. For example, the expression of the S protein has been confirmed by immunostaining with a FITC-labeled secondary antibody after transfection of Vero cells with ZyCoV-D candidate vaccine constructs [36]. In vitro studies with INO-4800 revealed the expression of the S protein at both the RNA and protein levels in COS-7 cells and HEK-293T cells by RT–PCR and Western blotting [37]. Similarly, the robust expression of prefusion conformation S protein has been detected by flow cytometry after the incubation of HEK293T/17 cells with BNT162b2 or mRNA-1273 [42].

### 2.3. Determining Toxicological Effects and Immune Effects in Animal Models

The toxicological effects of vaccines are the first priority to be considered before further research and are mainly assessed by the occurrence of adverse reactions, whereas immune effects are the most concerning issue throughout the world and are highly linked with immunogenicity. Immunogenicity is reflected by the intensity of both humoral and cellular immunity after vaccination. When the S protein is consistently synthesized, antigens are identified and presented by MHC-I and II, which then activate cytotoxic CD8 + T cells (MHC-I-mediated) and helper CD4 + T cells (MHC-II-mediated) and enhance memory T cell production [43,44]. CD8 + T cells can kill other infected or damaged cells. The activated CD4 + T cells release cytokines that induce B cells to divide into either plasma cells or memory B cells. Plasma cells secrete a large number of antibodies into the circulatory system, while memory B cells remain inactive. Once they encounter the same antigen, memory B cells rapidly divide into plasma cells in response to reinfection. Recovery from COVID-19 is associated with the generation of binding antibodies, as well as neutralizing antibodies that can neutralize viruses in recovered individuals [45,46], and the activation of T lymphocytes that can destroy pathogen-infected cells.

If safety and efficacy are not guaranteed, vaccination may not only fail to reach the prevention effects but also yield additional infection risks. Therefore, preliminary evaluation in animal trials is indispensable for further clinical experiments. To ensure reliability and reproducibility, animal experiments are rigorously conducted to good laboratory practice standards. During the development of COVID-19 vaccines, animal experiments are performed first, and the above nucleic acid vaccines are well tolerated without any serious safety issues in animal models, but further validation is needed in population groups. A preliminary animal study conducted in rats and rabbits demonstrated that ZyCoV-D induced an antibody response, including neutralizing antibodies against SARS-CoV-2, and elicited a Th1 response, as evidenced by elevated IFN-γ levels [36]. For INO-4800, functional antibodies and T cell responses were all measured in multiple animal models. Humoral immunogenicity testing in both mice and guinea pigs revealed that INO-4800 was capable of eliciting functional blocking antibody responses to the S protein in the serum and lungs, which was confirmed by measuring serum IgG binding endpoint titers, antibody neutralizing activity, and receptor inhibiting functionality. Strong T cell responses were found by an IFN-γ enzyme-linked immunospot assay (ELISpot). Subsequently, T cell populations producing IFN-γ have been identified, and T cell responses against S protein epitopes have been detected in immunized mice [37]. For BNT162b, B and T cell responses were characterized in a series of experiments in BALB/c mice and rhesus macaques after intramuscular immunization. Strong humoral immunogenicity was demonstrated by elevating RBD-specific IgG levels and increasing IgG affinity to RBD. A high fraction of CD4 + and CD8 + T cells that produced IFNγ and CD8 + cells that produced IL-2 have been confirmed by ELISpot or intracellular cytokine staining (ICS) flow cytometry analysis [47]. mRNA-1273 induced both potent neutralizing antibody and CD8 + T cell responses and protected against SARS-CoV-2 infection in the lungs and noses of mice [42]. Consistent with these results in nonhuman primates, vaccination of Indian-origin rhesus macaques induced robust neutralizing activity, rapid protection in the upper and lower airways, and no pathologic changes in the lung [48]. Collectively, the above nucleic acid vaccines are well tolerated and all elicited both humoral and cellular immunity in multiple animal models, which supports further clinical development.

### 2.4. Clinical Trials

Clinical trials are prospective biomedical or behavioral research studies on human participants designed to answer specific questions about biomedical interventions and new treatments and to determine whether new treatments are safe and effective [49,50]. Depending on the development stage, clinical trials may proceed through four phases from single research center, one country small and pilot studies, to multiple centers, multiple countries, and larger scale comparative studies [51]. Once a new treatment successfully passes through phases one–three, it will usually be approved by the national regulatory authority for use in the general population. Phase four trials are performed after a newly approved treatment is marketed, providing an assessment of the risks, benefits, or best uses [52]. Usually, clinical trials are costly and of a long duration, with low approval rates [53]. Only 10% of all drugs that start in human clinical trials become approved drugs.

The clinical trials of the COVID-19 vaccine candidates have followed the International Council for Harmonization of Technical Requirements for Pharmaceuticals for Human Use and Good Clinical Practice guidelines. They generate data on dosage, safety, and efficacy. The data collected and aggregated allow the investigators and regulatory agencies to monitor the aggregate use profile of experimental medicines. In many countries, adverse effects are required to be reported and researched in clinical trials by law, and reporting systems have been built and continuously refined, such as the Uppsala Monitoring Centre of the WHO, the European Medicines Agency (EMA) of the European Union, the Food and Drug Administration (FDA) of the United States, the National Medical Products Administration of China, and the Marketed Health Products Directorate of Health Canada of Canada.

#### 2.4.1. Dosage

The amount of drug to be administered to recipients is determined during clinical trials. For example, three doses of ZyCoV-D are administered intradermally via a needle-free injection system 28 days apart (2.0 mg/dose) [54]. The INO-4800 is administered intradermally by electroporation using the CELLECTRA^®^ 2000 device (Inovio Pharmaceuticals, Plymouth Meeting, PA, USA) in a two-dose regimen (2.0 mg/dose) at 0 and 4 weeks [55]. BNT162b2 is administered intramuscularly as a series of two 30 µg doses of the diluted vaccine solution (0.3 mL each) 21 days apart. mRNA-1273 (100 μg) is administered intramuscularly as a series of two 100 μg doses of the diluted vaccine solution (0.5 mL each) 28 days apart.

#### 2.4.2. Safety

Vaccination safety of COVID-19 vaccines in humans is mainly assessed by adverse reactions, which consist of immediate adverse reactions (within 30 min), solicited adverse reactions (within 7 days), unsolicited adverse reactions (within 28 days), and suspected unexpected serious adverse reactions [56]. Depending on the location, adverse reactions comprise local adverse reactions limited to a certain location and systemic adverse reactions causing adverse effects throughout the systemic circulation. Adverse reactions are graded according to a standard toxicity grading scale [57]. For COVID-19 vaccines, the most solicited common local adverse reactions include pain, redness, and swelling at the injection site, whereas the common systemic adverse reactions are fatigue, headache, fever, myalgia, diarrhea, nausea, cough, hypersensitivity, decreased appetite, etc. (Table 2). Serious adverse events are rare and typically involve anaphylactic reactions and thrombotic thrombocytopenia [58]. However, the exact causality has yet to be proven. Theoretically, nucleic acid vaccines are relatively safe and without atavistic risk due to containing partial pathogen genome sequences. However, the risk of foreign DNA integrating into the host chromosome is persistently present. Anaphylactic reaction and myocarditis have been reported more frequently after the administration of BNT162b2. Additional studies and prolonged observation periods are evidently needed. The safe characteristic features of nucleic acid vaccines are discussed in detail below.

The phase three clinical trial of ZyCoV-D at 49 centers in India demonstrated that most of the adverse events were mild or moderate in intensity [54]. No difference was observed with respect to successive dosing within each group or between the treatment groups. The most frequently reported local adverse event in both the ZyCoV-D groups and placebo groups was pain at the injection site, whereas the most commonly reported systemic adverse event in both treatment groups was headache. There were no deaths or serious adverse responses reported in the phase one clinical trial [59]. The phase three clinical trial reported 15 serious adverse events, including two fatal serious adverse events. However, none of them were considered causally related to the vaccine. The phase one clinical trial of INO-4800 showed that all adverse responses were Grade 1 (mild) in severity. The most commonly reported local adverse responses in participants were injection site pain and erythema, whereas the most commonly reported systemic adverse response was nausea. All related adverse responses occurred on the dosing day when the subjects received the first or second vaccination. No serious adverse events or adverse events of special interest were reported [55].

The multinational clinical trial of BNT162b2 and mRNA-1273 demonstrated that adverse reactions were reported more often by vaccine recipients than by placebo recipients, more often by younger vaccine recipients than by older vaccine recipients, and more often after dose two than dose one [60]. The most commonly reported local adverse response in participants was pain, whereas the most commonly reported systemic adverse responses were fatigue and headache. The severity was mild to moderate and usually resolved within 48 h [61]. Several adverse events of BNT162b2 have been reported in individuals in the vaccination group or placebo group and include shoulder injury, lymphadenopathy in the arm and neck region, Bell’s palsy, anaphylactic reactions, myocarditis, and pulmonary fibrosis [62]. Among these adverse events, the US Centers for Disease Control and Prevention identified 21 cases of serious anaphylaxis, corresponding to an estimated rate of 11.1 cases per million doses administered. However, 17 of 21 patients with anaphylaxis had a documented history of allergies or allergic reactions [63]. The adverse events of mRNA-1273 included lymphadenopathy, Bell’s palsy, and anaphylactic reactions. Healthy young individuals definitively diagnosed with myocarditis after receiving the second dose of the mRNA vaccine (BNT162b2 or mRNA-1273) have been reported one after another [64,65]. Similar findings of a more pronounced risk of myocarditis after mRNA-1273 in comparison with BNT162b2 have been observed in other large observational studies [66,67]. Further mechanistic studies are therefore warranted and could provide valuable insight. Meanwhile, some sporadic articles have also reported that vaccinated individuals developed myocardial microthrombi [68] and acute exacerbation of idiopathic pulmonary fibrosis [69]. Together, the adverse reactions are not merely correlated with the nucleic acid but are also related to other components and the physical fitness of recipients.

#### 2.4.3. Effectiveness and Immunogenicity

Simulation experiments have revealed that to prevent an epidemic, vaccine efficacy must be at least 60% when vaccination coverage is 100%. This vaccine efficacy threshold rises to 70% when coverage drops to 75% and up to 80% when coverage drops to 60% [70]. Vaccine efficacy is assessed by comparing the percentage of reduction in disease incidence in a vaccinated versus unvaccinated population, which is closely related to humoral and cellular immune responses in vaccine recipients [71]. In clinical experiments, the common indicators used to evaluate humoral immunity include seroconversion rates based on neutralizing antibody amounts and the geometric mean titers (GMTs) of specific antibodies [72,73]. Seroconversion in COVID-19 patients refers to the development of specific antibodies in the blood serum against the S antigen or N antigen, which is defined as antibody-negative subjects at baseline who become antibody-positive after vaccination and subjects having antibody titers at baseline who have a fourfold rise in antibody titers after vaccination [74]. The higher the rate of seroconversion, the more protective the vaccine for a greater proportion of the population. The cellular immune response is used to detect the contribution of CD4 + and CD8 + T cells after vaccination, such as cytokine secretion of IFN-γ, interleukin-2 (IL-2), and tumor necrosis factor α (TNF-α), which are measured by both IFN-γ ELISpot and ICS [75,76].

Clinical trials have demonstrated that the efficacy of three doses of ZyCoV-D was 66.6% against symptomatic COVID-19 among participants 12 years of age or older after administration into the skin using a needle-free device [54]. During the Delta variant pandemic, the efficacy was essentially against this variant [28]. The trial is still underway, and late-stage trial results have yet to be fully published. The multinational study showed that BNT162b2 was 95.0% effective in preventing COVID-19 at least 7 days after the second dose among participants 16 years of age or older. Similar efficacy levels were observed across subgroups defined by age, sex, race, ethnicity, baseline body mass index, and the presence of coexisting conditions [62]. Real-world surveillance in Israel revealed that two doses of BNT162b2 were highly effective in preventing symptomatic and asymptomatic SARS-CoV-2 infections and COVID-19-related hospitalizations and death [77]. For mRNA-1273, the phase three randomized trial conducted at 99 centers across the United States demonstrated that vaccine efficacy was estimated at 94.1% in preventing COVID-19 at least 14 days after the second injection among participants 18 years of age or older. Efficacy was similar across key secondary analyses, including assessment 14 days after the first dose, analyses that included participants who had evidence of SARS-CoV-2 infection at baseline, and analyses of participants 65 years of age or older [60]. Efficacy against severe COVID-19 was also high, with all 30 cases occurring 14 or more days after the second dose being in the placebo group. For the Delta variant, the efficacy levels of BNT162b2 and mRNA-1273 were approximately 40% and 75%, respectively [78,79], which might be associated with the stronger Fc-mediated effector functions of mRNA-1273 [80]. However, these findings demonstrate that three-doses of mRNA-1273 had lower effectiveness against Omicron infection, particularly among immunocompromised people [81].

In the phase one trial of ZyCoV-D, seroconversion based on humoral responses was observed in 80% of participants who received three doses of 2 mg vaccine via a needle-free injection system. Vaccination resulted in a 10–12-fold rise in IFN-γ spot-forming cells per million peripheral blood mononuclear cells, suggesting a strong cellular response [59]. The immunogenicity response seen in phase one/two was maintained in the phase three study, as well [54]. In the phase one trial of INO-4800, all subjects evaluable for immunogenicity had cellular and humoral immune responses following the second dose. Overall seroconversions in the 1.0 mg and 2.0 mg dose groups were 95% for each group. Vaccination led to substantial T cell responses with an increased Th1 phenotype, as evidenced by the increased expression of the Th1-type cytokines IFN-γ, TNF-α, and IL-2. Importantly, this was accomplished while minimizing the induction of IL-4, a prototypical Th2 cytokine. The study of INO-4800’s efficacy has now entered late-stage trials [55]. According to the phase one/two trial conducted in the United States [82], BNT162b2 elicited a strong humoral immune response (GMTs are up to 3.3-fold above those samples from individuals recovered from COVID-19) at one week after the booster and a strong cellular immune response (a strong response of IFNγ+ or IL-2 + CD8 + and CD4 + type 1 helper T (Th1) cells against epitopes that are conserved in a broad range of variants) throughout the full observation period of nine weeks following the booster [83]. The GMTs peaked one week after the second vaccination and began decaying one week after that. For mRNA-1273, after the second vaccination, binding-antibody responses reached the upper quarter of the distribution of responses among the controls who donated convalescent serum. The GMTs of the participants far exceeded the responses among participants who donated convalescent serum. The vaccine elicited a CD4 + T cytokine response involving Th1 cells (a strong response of TNF-α, IL-2, and IFNγ) and type 2 helper T (Th2) cells (a minimal response of IL-4 and IL-13) among participants. CD8 + T cell responses were observed among the participants only at low levels after the second vaccination [61].

Although the efficacy of ZyCoV-D seems to be lower than that of the mRNA vaccines, the figures are not comparable. The ZyCoV-D trials in India earlier this year were conducted while the Delta variant was the dominant variant, whereas the earlier mRNA vaccine trials were conducted when less transmissible variants were circulating [28]. The above nucleic acid vaccines all elicit either humoral or cellular immune responses. Both BNT162b2 and mRNA-1273 induce robust functional humoral immune responses, with differences in epitope recognition and antibody-mediated functional properties. Compared to BNT162b2, mRNA-1273 induces higher concentrations of RBD- and N-terminal domain-specific IgA and elicits stronger neutrophil phagocytosis and natural killer cell activation [80].

## 3. The Pros and Cons of Nucleic Acid Vaccines

### 3.1. Comparison of Nucleic Acid Vaccines and Traditional Vaccines

Nucleic acid-based vaccines with theoretical advantages over conventional vaccines are attractive platforms with great opportunities and challenges. Compared to traditional vaccines, nucleic acid vaccines present several advantages. The major one is that the target genes can be anthropogenic modifications based on the dominant antigenic epitopes. Once new virus variants occur, targeted vaccines can be prepared rapidly and inexpensively on a large scale, which is essential for controlling an unexpected epidemic outbreak. Due to the singleness of antigen components, the phenomenon of antibody-dependent enhancement (ADE) is rare in nucleic acid-based vaccines [84]. As a consequence of containing partial, but not all, pathogen genome sequences, atavistic risk is absent. Moreover, nucleic acid vaccines can induce strong and long-lasting humoral and cellular immune responses simultaneously. It has been demonstrated that foreign plasmid DNA can still be detected by PCR in mice at 15 months after intramuscular injection [85].

Nucleic acid-based vaccines are highly promising. However, their development is nascent, and much remains to be further validated, such as safety. Synthetic raw materials and encrusting materials are likely to be toxic, presenting the risk of peripheral host cell damage. For example, it has been reported that the polyethylene glycol (PEG) used to conjugate lipids in mRNA vaccines is associated with anaphylaxis events [86]. The nucleic acid persisting in vivo contributes to the production of self-reactive antibodies and then induces autoimmune disease. Some patients with common autoimmune diseases are detected to have more than one anti-nucleic acid autoantibody, such as systemic lupus erythematosus [87], multiple sclerosis [88], rheumatoid arthritis [89], and polymyositis [90]. With rapid development, growing safety concerns are particularly apparent for DNA vaccines. This foreign DNA is likely to integrate randomly into the host chromosome, thereby leading to the activation of oncogenes, inactivation of tumor suppressor genes, or other chromosomal instability.

### 3.2. Comparison of DNA Vaccines and RNA Vaccines

Although similar in many ways, there are some important distinctions between DNA vaccines and RNA vaccines. First, the inoculation means are different in that DNA vaccines do not exert their function until they reach the nucleus, whereas RNA vaccines only need to enter the cytoplasm. Therefore, DNA vaccines struggle to induce potent immune responses in clinical trials after intramuscular vaccination, which is why research on their clinical application is progressing slowly. Many vaccination methods with gene guns [91] or electroporation apparatus [92] are now available; however, they are costly and experimentally more challenging. They all limit the application of DNA vaccines. Second, the risk of vaccination varies between DNA and RNA vaccines. RNA vaccines both preserve the advantages of intracellular expression of target antigens and overcome potential risks of integrating into the host DNA. In addition, their stability in vitro is different. DNA with a unique double-helix structure is strongly associated with good stability and an extended storage period, whereas mRNA is easily catabolized by host machinery under the physiologic conditions of ubiquitous ribonucleases. For example, the RNA vaccine of BNT162b2 requires storage at −70 °C, which introduces additional burdens for vaccine distribution and transportation.

## 4. Optimization

Nucleic acid vaccines have many unique advantages, but there are many aspects that require optimization. The selection of target genes is of particular importance during the development of nucleic acid vaccines. It has been reported that the different target genes cause the immune effects to be very different [93]. A series of sequence candidates expressing different forms of the S protein have been evaluated in rhesus macaques, and the results confirm that the vaccine encoding the full-length S protein is superior to others in overall immune effects [94]. For DNA vaccines, plasmids must contain a promoter with strong transcriptional activity and a terminator with a strong termination signal. Plasmids containing sequences that are homologous to host cellular genes or easily integrated into host cells should be avoided. Delivery systems may need further optimization to improve delivery efficiency and safety. A study found that the polyethylene glycol used to conjugate lipids in mRNA vaccines is associated with anaphylaxis events [86]. Vaccination strategies are also an important aspect requiring attention, which is critical to boost immune responses while reducing the number and dose of vaccinations. Compared with homologous strategies, heterologous prime-boost immunization with different classes of vaccines improves both humoral and cellular immune responses, which has been confirmed in an animal model [95]. DNA vaccines and RNA vaccines are both incapable of activating the mucosal immune response, and aerosolized boosters may be useful solutions. Therefore, how to further improve multiple immune responses warrants further investigation.

## 5. Conclusions

Although the concept of nucleic acid vaccines has been proposed for many years, they are developing slowly because of their low delivery efficiency. Meanwhile, because previous outbreaks are well controlled in the short term, large population-based trials could not be performed, which severely hinders the research process. The COVID-19 outbreak has accelerated the development of nucleic acid vaccines, and several vaccines have been authorized successively by the WHO in a short duration of time. However, the development is nascent, and the long-term safety and efficacy need to be further evaluated. Currently, the largest concern regarding DNA vaccines is still their safety, such as whether viral DNA inserts the genome of the host or induces anti-nucleic acid autoantibodies. The highlight of RNA vaccines is their high effectiveness, but their poor stability is a key issue that needs to be addressed. Overall, nucleic acid vaccines with tremendous application prospects are indispensable in controlling a sudden pandemic. This is a very important step forward in the fight to defeat outbreaks globally because it demonstrates that we have another class of vaccines that we can use.

## Figures and Tables

**Table 1:** The current COVID-19 nucleic acid vaccines and details.

Vaccine Name	Technology	Developer/Company	Immunization Protocol	Immunity	Effectiveness	Current Status
ZyCoV-D	DNA vaccine	Zydus Cadila (Ahmedabad, India)	3 doses (2.0 mg/dose), 4 weeks apart	humoral and cellular immunity	66.6%	approved by India
INO-4800	DNA vaccine	Inovio (Plymouth Meeting, PA, USA)	2 doses (2.0 mg/dose), 4 weeks apart	humoral and cellular immunity	unpublished results	phase Ⅱ/Ⅲ clinical trials
AG0302-COVID19	DNA vaccine	AnGes (Osaka, Japan)	2 doses (2.0 mg/dose), 2/4 weeks apart	unpublished results	unpublished results	phase Ⅱ/Ⅲ clinical trials
GX-19N	DNA vaccine	Genexine (Seoul, Korea)	unpublished results	unpublished results	unpublished results	phase Ⅱ/Ⅲ clinical trials
BNT162b2	mRNA vaccine	Pfizer (New York, NY, USA)/BioNTech (Mainz, Germany)	2 doses (30 μg/0.3 mL/dose), 3 weeks apart	humoral and cellular immunity	95.0%	approved by WHO
mRNA-1273	mRNA vaccine	Moderna (Cambridge, MA, USA)	2 doses (100 μg/0.5 mL/dose), 28 days apart	humoral and cellular immunity	94.1%	approved by WHO
ARCoV	mRNA vaccine	WALVAX (Yunnan, China)/ABOGEN (Suzhou, China)	unpublished results	humoral and cellular immunity	unpublished results	phase Ⅱ/Ⅲ clinical trials

**Table 2:** The common adverse reactions to COVID-19 nucleic acid vaccines.

Vaccine Name	Common Local Adverse Reactions	Common Systemic Adverse Reactions	Serious Adverse Events
ZyCoV-D	pain, redness, swelling, and itching	headache, fever, muscle pain, and fatigue	cerebrovascular stroke, cardiorespiratory arrest with septicaemia, and alcoholic liver disease
INO-4800	pain and erythema	nausea	none currently reported
BNT162b2	pain, redness, and swelling	fatigue, headache, chills, and muscle pain	hypersensitivity reaction, paroxysmal ventricular arrhythmia, and death
mRNA-1273	pain, erythema, swelling, and lymphadenopathy	fatigue, headache, myalgia, and arthralgia	hypersensitivity reactions, Bell’s palsy, and death

## Data Availability

The data that support the findings of this study are available from the corresponding author upon reasonable request.
